# Pathology

**Published:** 1890-11-08

**Authors:** 


### PATHOLOGY.

Dr. Savill first showed the specimens and described the appearances of the genito-urinary apparatus from a man, J.M., aged 61, who presented some interesting features. Consequent upon a stricture in early life the walls of the bladder had become much thickened, and the trabeculæ enlarged; the ureters were dilated, so also the pelvis of the kidneys, and the glands themselves presented the characters of surgical kidneys. Although the man died from pulmonary tuberculosis there was no evidence of tubercle in the kidneys. The next case described was that of a woman, M. W., 99 years of age. Family history: her father died, aged 85, from dry gangrene, her mother died at 93, one brother at 85, and two more brothers at 75 and 81 years respectively. There had been thirteen in the family, and three had died from phthisis in middle life. Three of her own children had also died from phthisis. She had enjoyed fair health up to 50 years old; at this time she was said to have had dropsy all over, including her face, and this was followed by eczema. At one time she is stated to have suffered from meningitis. Seven years before her admission into the infirmary she suffered from general weakness, and often fell down; two years before her death she fell and fractured her hip. On admission her mental condition was described as follows: "She forgets recent events, forgets where she is, the day of the week or month, but recollects many events of her earlier life, and even pieces of poetry. Thus she presented the most characteristic condition of the senile mental state. Another point observed by Dr. Savill in old people, namely, the tendency to harp on one idea; to be, for instance, always asking the same question, and this, which he believed was often the earliest symptom of senile mental change, was also present. On admission the patient was bed-ridden, and showed the result of seven years wasting of the muscles and loss of power of movement, together with sallowness of the skin; the urine was clear, pale, acid, 1,006, no albumen, and there was no frequency of micturition. Soon afterwards she became drowsy, in fact her time appeared to be divided between a drowsy semi-conscious condition, and one of rambling incoherence; towards the end there was some œdema of the legs, and she died from asthenia. Post-mortem: There was slight œdema of the ankles. The heart presented some puckering of the mitral valve, but there was no hypertrophy of the left ventricle, and under the microscope there was seen some brown atrophy of the muscular substance. The arteries, both the arch and the descending aorta showed advanced atheromatous change, especially marked in the abdominal aorta which was calcareous, the arteries of the brain were also atheromatcus, and the brachial artery presented some thickening and increase in its muscular coats. The lungs revealed brown induration and fibrosis, and some commencing pneumonia. The bases were congested, and the edges emphysematous. Both apices presented some cicatricial tissue, with slight puckering of the surface, apparently obsolete tubercle. The liver showed the folds on its surface said to be produced by tight lacing in early life, and weighed 22½ ounces; there was slight cirrhosis, but the substance was soft, and under the microscope showed early fatty changes. The kidneys were granular, each weighing 2½ ounces, the capsules thick and adherent, the surface beneath being granular; on section they showed considerable decrease in the cortical region, which was only about one-sixth or one-seventh the thickness of the medullaty part; the substance was dark coloured, and the vessels stood out prominently. Under the microscope they showed infiltration, and in places obliteration of the tubules. The brain had an excess of serum on the surface—"serous apoplexy" of old writers. After describing these two cases, Dr. Savill discussed kidney disease in general and chronic Bright's disease in particular. He defined chronic Bright's disease, or contracted granular kidney, as a chronic degeneration of the kidney substance with a tendency to albumen, dropsy, and uræmia. In 1802, Dr. Wells first found albumen in cases of dropsy. Soon after this Blackall divided dropsies into those with, and those without albumen, but attributed the albumen to the influence of the lymphatics. In 1836, Bright, in a paper published in the Guy's Hospital Reports, connected dropsy and albuminuria with kidney disease. The varieties of Bright's disease have been much confused owing to two causes—(1) many anatomical distinctions have been made by pathologists which have no corresponding clinical features; and (2) they have introduced a multiplicity of names, and hence much complicated the subject. The following is a convenient summary of kidney diseases: (*a*) Congestions (cardiac kidney), with an increase of the supporting stroma; (*b*) acute inflammatory—(1) pyelitis, (2) acute nephritis (acute Bright's disease); (*c*) chronic degeneration (chronic Bright's disease); (*d*) benign and malignant growths—embolism, cancer, villous disease, hydatid; (*e*) hæmaturia and parasites—(1) paryoxymal hæmaturia, (2) endemic hæmaturia and Bilharzia; and (3) chyluria. Clinically, diseases of the kidney may be divided into three groups, the main pathological feature of each group corresponding with morbid changes in one of the three main structures of the organ—the parenchyma, the stroma, or the vessels. (*a*) The smooth, or large white kidney, starts as an acute nephritis; in the second stage the kidney is enlarged, the increase of substance being essentially in the cortex; the organ is soft, pale, opaque, the capsule readily strips, the organ is anæmic, except in the region of the cones and on the surface. In the third stage there is frequently atrophy and contraction of the tubules, and the capsule may be slightly adherent. (*b*) The gouty kidney is contracted from the very first, fluid and cells being thrown out between the tubules, these produce an hypertrophy of the stroma, the capsule becomes adherent, and leaves beneath a granular appearance when removed. On section the gland is of a reddish brown colour, very firm, with contraction of the cortex, and the vessels stand out prominently. (*c*) The waxy or amyloid kidney; in the first stage the organ is not much altered in size, but presents the glistening appearance of the malphigian bodies on section. In the second stage it is enlarged, denser, and harder, the capsule is not adherent. In the third stage it is contracted again to about its normal size. In a still later stage there may be some deposit of interstitial tissue, and so there may be some adherence of the capsule. In addition to these two well marked kinds, a mixture of *a* and *b*, or of *a* and *c* may be found. What is known as the post-scarlatinal kidney, differs from the large white in some special features, such as a less tendency to fat in the cells of the tubules, but a deposit of lime salts in the cells, and a thickening of the muscle in the walls of the arteries.

